# TECS: a toxin expression control strategy as a tool for optimization of inducible promoters

**DOI:** 10.1186/s12934-018-0891-1

**Published:** 2018-03-13

**Authors:** Aleksandra Małachowska, Paweł Olszewski

**Affiliations:** Department of Genetics and Biosystematics, University of Gdańsk, ul. Wita Stwosza 59, 80-308 Gdańsk, Poland

**Keywords:** Inducible promoter, Promoter engineering, Promoter optimization, Conditional toxicity, Promoter library

## Abstract

**Background:**

Transcriptional control of gene expression is a widely utilized regulatory mechanism in synthetic biology, biotechnology and recombinant protein production. It is achieved by utilization of naturally occurring promoters responding to nutrients or chemicals. Despite their regulatory properties, these promoters often possess features which diminish their utility for biotechnology. High basal expression level and low induction ratio can be removed using genetic engineering techniques, although this process is often laborious and time-consuming.

**Results:**

In order to facilitate optimization process for inducible promoters, we developed a simple method based on a conditional toxin expression which we abbreviate as toxin expression control strategy (TECS). In the presence of sucrose, SacB enzyme from *Bacillus subtilis* synthesizes levans which cause *Eschericha coli* cell lysis. However, in the absence of sucrose the enzyme does not affect the growth of the host. We utilized this feature to develop a two-step protocol allowing for efficient selection of inducible promoter variants. Using TECS we were able to modify the well-described p_BAD_ promoter to decrease its leakage while maintaining high activity upon induction. Furthermore, we used the method to test transcriptional interference of lambda phage-derived sequence and optimize it for higher induction levels through random mutagenesis.

**Conclusions:**

We show that TECS is an efficient tool for optimization and development of inducible promoter systems in *E. coli*. Our strategy is very effective in the selection of promoter variants with improved properties. Its simplicity and short hands-on time make it an attractive method to optimize existing promoters and to construct novel, engineered genetic elements which improve properties of an inducible promoter system.

**Electronic supplementary material:**

The online version of this article (10.1186/s12934-018-0891-1) contains supplementary material, which is available to authorized users.

## Introduction

Biotech markets are among the fastest developing markets worldwide [1]. One of the reasons for this development is growing demand for recombinant protein production which is widely used in science, industry and medicine [2]. To date, the most efficient way to produce proteins is to use living organisms as factories. However, achieving a high yield of a recombinant protein relies on multiple factors, among which the most important may be the choice of the appropriate inducible promoter [3, 4]. Despite numerous available inducible promoters, there is no universal system [5]. Thus finding an optimal inducible promoter system often requires a time-consuming trial and error period and to our knowledge, there are no simple tools allowing for optimization and generation of custom inducible promoters [6].

There are several, well-studied models of inducible promoters among which lactose-inducible p_tac_ and l-arabinose-inducible p_BAD_ are the most frequently used [5]. Both promoters are derived from *Escherichia coli* in which they regulate lactose operon and arabinose operon expression respectively [4, 6]. The p_tac_ promoter is a flagship example of an inducible promoter engineering since the modern version is a highly modified version of p_lac_ promoter of *E. coli*. The original p_lac_ promoter, in spite of being regulated by lactose, was dependent on cAMP levels and had low intrinsic activity [7]. In the course of engineering, the *lacUV5* mutant was selected which had decreased dependency on cAMP [8]. Next, in order to improve induction level, a hybrid with strong p_trp_ promoter was created. This resulted in over tenfold increased induction ratio with respect to the wild-type promoter [9]. However, despite these improvements, p_tac_ promoter is rarely used directly for recombinant protein production. Instead, it is used to control T7 polymerase expression which drives the expression of a target gene under the control of the T7 promoter in pET series of expression vectors [10].

In contrast to the p_tac_, the p_BAD_ promoter used in biotechnology is the unmodified version of *E. coli* promoter. In general, it has similar induction level as p_tac_ promoter and all-or-none induction profile what does not allow for gradual induction level [10, 11]. Nonetheless, it has a major advantage over other promoters which is a tight regulation of expression, resulting in low basal expression levels in the absence of the inducer, making it suitable for the production of moderately toxic proteins like membrane proteins [12]. Since p_BAD_ promoter offers remarkably robust gene expression regulation, efforts were made to eliminate all-or-none induction profile and increase the strength of the promoter. However, it was shown that interference with promoter sequence or flanking sequences results in alleviation of AraC-mediated regulation of expression or decreased activity of the p_BAD_ promoter [13]. Therefore, researchers focused on the elimination of other features like all-or-none induction phenomenon, which was bypassed by constitutive expression of *araE* encoding arabinose transporter [14] or engineering the repressor-activator protein AraC [15, 16]. Nevertheless, despite successful engineering of repressor-activator protein, the commonly used pBAD24 vector bears the wild-type version of the *araC*-p_BAD_ sequence.

There are two main approaches used in the promoter engineering process. One of them is the rational design, which relies on the available knowledge about output provided by particular changes to the promoter sequence [17]. Mutations to be introduced are selected on the basis of characterized promoters with a defined output or on the basis of computationally determined weight matrices [18–20]. The most recognized example of the rational design in promoter engineering is the aforementioned p_tac_ promoter. The second approach is to generate a randomized or synthetic promoter library (SPL), either through oligonucleotide introducing random sequences between core promoter elements, or through mutagenic PCR [21]. Furthermore, by using fluorescent protein as a reporter and fluorescence activated cell sorting (FACS) it is possible to generate SPL’s with broad range of promoter activities [15, 22, 23]. Promoter randomization in combination with FACS is frequently used for constitutive SPLs but there are also examples of random library generation for an inducible promoter in bacteria [24, 25]. Although successful, these approaches often yield inducible promoters with higher level of background expression and might have relatively low efficiency (less than 2% of clones selected [25]).

The major obstacle in engineering inducible promoters is the need to preserve regulatory properties of the promoter namely low leakage level and activation by the inducer. These requirements complicate the process of selection hindering the use of high throughput technologies (FACS) which leads to the laborious testing of individual clones. We found that using a conditionally toxic gene as a reporter can be successfully utilized for selection of inducible promoters. In this work we show that our approach is efficient in modification of an inducible promoter as well as in testing effects of exogenous regulatory sequence addition and its modification. We show that TECS allows for efficient selection of inducible promoter variants with various expression levels form relatively low number of clones. In addition to these features, the protocol presented in this work requires only basic microbiological equipment and simple procedures, making it an attractive choice for generation of custom, inducible promoter libraries.

## Materials and methods

### Bacterial strains, plasmids and media

Plasmids and primers used in this study are listed in Additional file 1: Tables S1, S2 respectively. All plasmids are available upon request. The ancestor plasmid for constructs used in this study is pBAD24 plasmid bearing *araC*-p_BAD_ sequence, pBR322 *ori* and ampicillin resistance [26]. pBAD24 cm is its direct derivative in which the coding sequence for beta lactamase (*bla*) was replaced with chloramphenicol acetyltransferase (*cat*) sequence. Remaining plasmids are derivatives of pBAD24 cm and were constructed through overlap extension PCR [27]. The *cat* gene was amplified from pACYC184 plasmid (ATCC 37033) with primers cat_overlap_fw and cat_overlap_rev. The *sacB* gene was amplified from the pKOV plasmid (Addgene 25769) with primers sac2bad_fw and sac2bad_rev. GFP sequence was amplified from pEGFP plasmid with primers gfp2bad_fw and gfp2bad rev. Sequence of relevant regions was confirmed by Sanger sequencing with pBAD_seq2 primer (Macrogen Europe). All experiments were performed in *E. coli* DH5α (Stratagene, La Jolla, CA, USA). For standard growth LB Miller liquid and solid medium was used (BioShop Canada Inc., Canada). For plasmid isolation cultures were grown in terrific broth (BioShop Canada Inc., Canada). Minimal medium was composed of M9 salts, 0.2% glucose, 0.2% casamino acids and 1 mM thiamine.

### Molecular cloning procedures

All cloning procedures as well as phenotype analyses were performed in DH5α strain. Competent cells were prepared with Inoue method [28]. Routine transformation protocol included 20 min incubation with DNA and 60–90 s heat-shock at 42 °C, followed by 1 h recovery in TB medium and plating. Standard transformations were plated on LB agar plates supplemented with chloramphenicol (Cm, 32 μg/ml). In the case of selection protocol, transformations were plated on LB-Cm plates with 5% (w/v) sucrose (the first selection step).

### PCR assembly and cloning of the p_O_-*oopRNA* fragment

The p_O_-*oopRNA* fragment was assembled from five oligonucleotides (oligo1-5, Additional file 1: Table S2) using the PCR-based assembly [29] (Additional file 1: Figure S1a). For assembly and cloning NEB Q5 Hot Start DNA polymerase was used (New England Biolabs, Ipswich, Massachusetts, USA). Overlaps between oligonucleotides were set to be close to 60 °C which was calculated in NEB Tm calculator. The final PCR product was used in the overlap PCR reaction with p_BAD_-sacB plasmid as a template using guidelines described in the original protocol [27]. 1 to 3 μl of the overlap PCR were used for bacterial transformation. For clone testing and sequencing, plasmids were isolated using one-tube protocol [30].

### Randomization of selected elements of the p_O_ promoter

Introduction of randomized sequence in − 10 and − 35 hexamers was performed with primer pairs 10_mut – 10_rev and 35_mut – 35_rev respectively (primer sequences are in Additional file 1: Table S2). The scheme of mutagenesis is presented in Additional file 1: Figure S1b. For the introduction of a 35 nucleotides-long random sequence substituting the p_O_ promoter, we used a procedure consisting of PCR with phosphorylated pomut1–pomut2 primers, self-ligation of the product and transformation (Additional file 1: Figure S1c). In detail, PCR primers were designed with phosphorylated 5′ end and randomized 5′ overhang (15–20 N nucleotides; Sigma-Aldrich). Vector amplification was performed with randomized primers and Hybrid DNA polymerase (EurX, Gdansk, Poland), which provided the highest yield of PCR product. Amplified DNA was precipitated by addition of equal volume of PEG solution (15% PEG, 1.25 M NaCl) and resuspended in nuclease free water. 20 ng were ligated with T4 DNA ligase (EurX, Gdansk, Poland) for 1 h at 37 °C in 100 μl volume to enhance self-ligation of the vector (EurX T4 DNA ligase manual). Subsequently, the reaction was precipitated, resuspended in water and used for transformation.

### GFP fluorescence assays

All measurements were carried out in 96-well, polystyrene plates. For standard measurements, the overnight cultures were diluted 1:50 in M9 minimal media supplemented with glucose, or other media depending on the experiment. GFP fluorescence was measured on EnSpire multimode reader (Perkin Elmer) at 488 nm excitation and 510 nm emission wavelengths. Cell density was measured by absorbance at 595 nm wavelength. Results of GFP intensity were represented as a ratio of GFP fluorescence to the culture density. All experiments were performed in at least three biological replicates. Significance of differences in GFP relative fluorescence between mutants and the wild type p_BAD_ promoter was tested with t test.

## Results

### Principle of TECS

Conceptual backbone of TECS is the utilization of toxic protein as an in vivo, self-selection factor. The strategy is based on three, simple assumptions: (i) basal expression or promoter leakage will result in the toxin production and growth impairment during the first selection step; (ii) mutations enhancing regulatory properties will decrease the leakage thus prevent toxin production and allowing normal growth; (iii) enhanced regulatory properties are valid only if leakage repression can be bypassed by the induction of the promoter, which would lead to toxin production and growth inhibition.

**Fig. 1:**
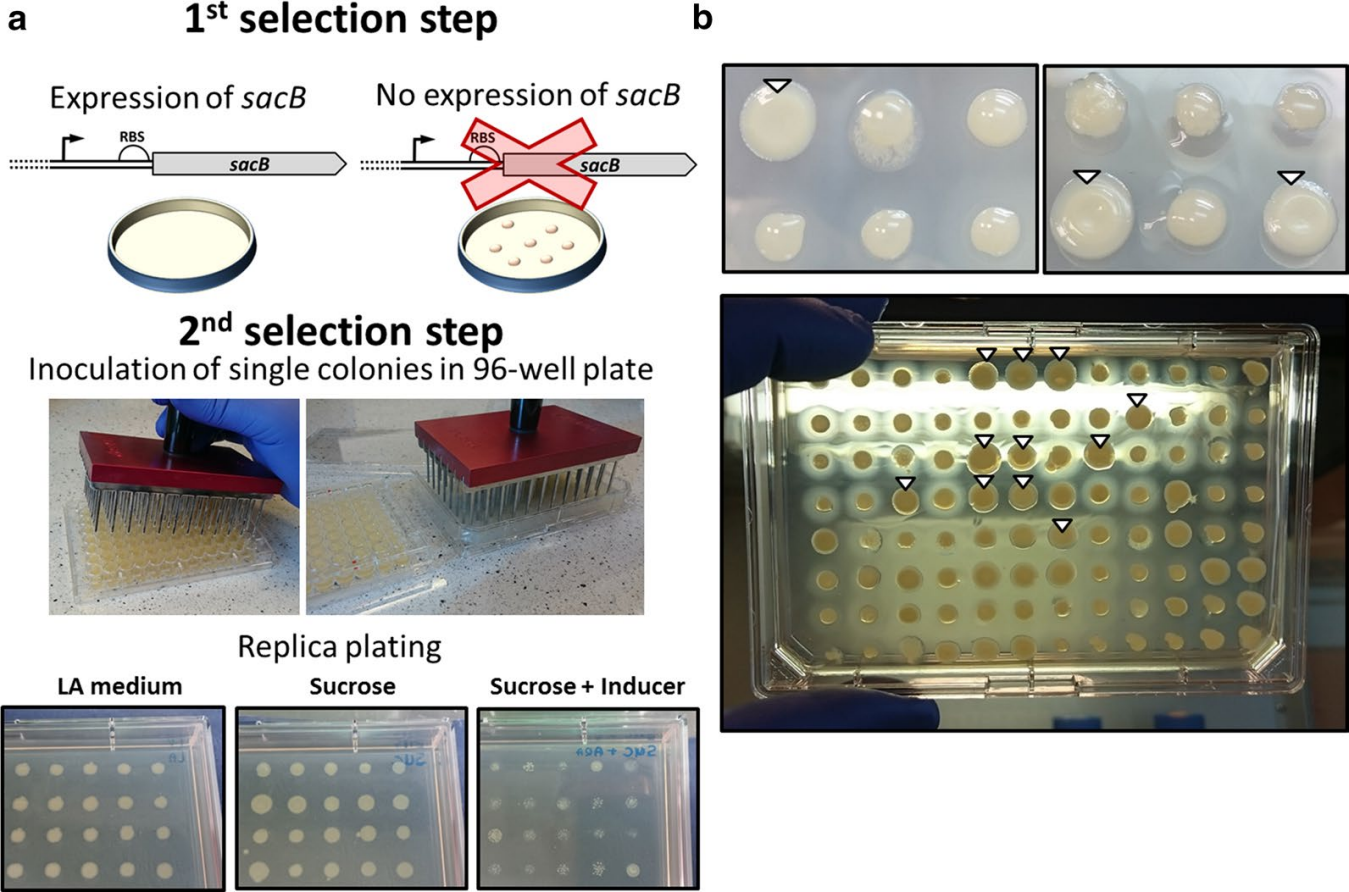
Scheme of TECS concept and two-step selection protocol. **a** TECS is based on the two-step selection process in order to select inducible promoter variants. An inducible promoter is placed upstream of *sacB* gene and used as the template for subsequent mutagenesis or randomization (the library of mutants). In the first step, the transformed library of promoter mutants is plated on 5% sucrose plates, on which only mutants with low or no expression of *sacB* will grow. Next, colonies are inoculated in a 96-well plate, grown over night and replica plated on trays with LA, sucrose or sucrose plus inducer media. **b** Phenotypes of cells producing SacB can be easily distinguished from non-producing colonies (marked by white triangles). High level of SacB results in lysis of cells and characteristic colony morphology. In addition, the presence of active SacB is manifested by white halo surrounding colonies

Empirical verification of these assumptions required a toxin, which toxicity can be additionally controlled in order to prevent killing the host. We chose to use SacB, which is a levansucrase producing levans from sucrose, which are toxic to *E. coli* [31, 32]. The conditional toxicity of SacB made it a perfect candidate for our protocol since in the absence of sucrose cells show no growth defects. Next, we developed a simple and robust protocol for the two-step selection of promoter mutants (Fig. 1a). The main purpose of TECS is to select best candidate promoters from a pool of randomized promoter variants (random promoter library), which can be generated by mutagenic PCR, site directed mutagenesis with random sequence-containing primers or cloning of a random sequence block. In the first step, mutated promoter library is transformed into *E. coli* and plated on media with 5% sucrose and resistance marker compatible with the vector. Fast growing, putative SacB-negative colonies are inoculated into a 96-well plate and replica-plated on selective media with sucrose and inducer (Fig. 1a middle panel). By comparing growth and colony morphology in the presence and absence of the inducer, it is easy to discriminate between clones producing SacB upon induction (Fig. 1b upper panel). In addition, further confirmation of SacB activity can be made after approximately 3 days of incubation, when a white halo is visible around colonies which produce the protein (Fig. 1b lower panel).

### Proof of concept

**Fig. 2:**
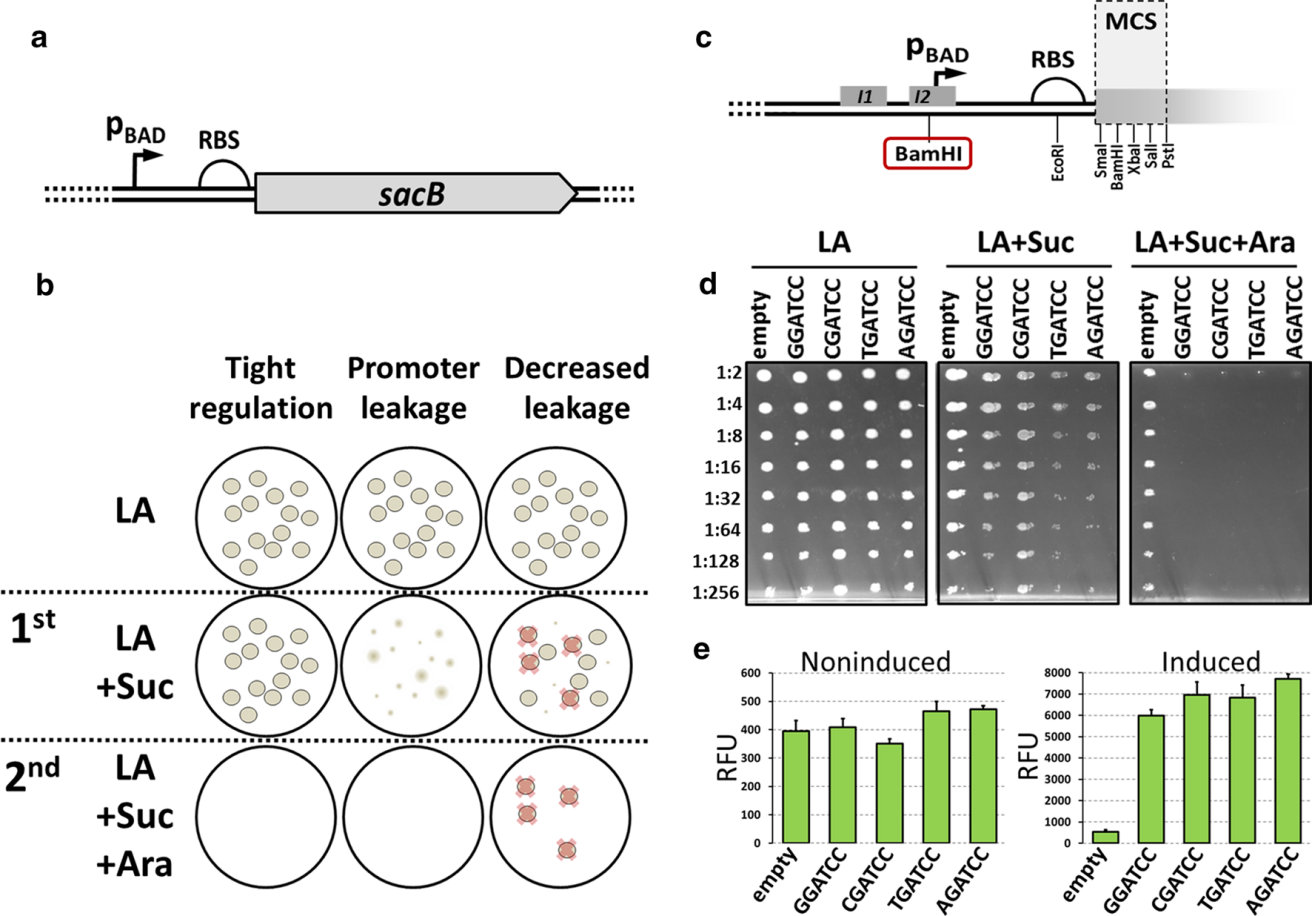
The concept and theoretical assumptions of TECS. **a** Schematic representation of the construct used to modify p_BAD_ promoter which regulates expression of *sacB* gene. The *Bam*HI site subjected to mutagenesis is circled. The underlined nucleotide in *Bam*HI recognized sequence was subjected to mutagenesis. **b** Graphical representation of theoretical assumptions of TECS. **c** Growth of indicated mutants of the *Bam*HI site. Two-fold dilutions of each mutant culture were plated on standard medium (LA), 5% sucrose (LA + Suc) and 5% sucrose plus 0.1% arabinose (LA + Suc + Ara). **d** Activity of mutated promoters measured with GFP fluorescence

As a model to test assumptions of TECS and to show its usefulness in the optimization of an inducible promoter, we chose a well-characterized p_BAD_ promoter which was placed in control of *sacB* gene expression (Fig. 2a) [26]. According to the model, the perfect regulation would result in normal growth on LA medium containing sucrose and no growth in medium containing sucrose and arabinose (induced condition) (Fig. 2b). However, even in the case of tightly regulated p_BAD_ promoter, leakage results in growth impairment in the presence of sucrose (Fig. 2b and c). The pBAD24 vector contains two *Bam*HI restriction sites, one located in the MCS and the other site is located in the *I2* operator site, which is bound by AraC protein in the presence of arabinose (Fig. 2a) [33]. The presence of the *Bam*HI site outside MCS is inconvenient for cloning with restriction enzymes but it was shown that the deletions or mutations within this site affects the activity of the p_BAD_ promoter [13]. With our protocol we tested effects of single nucleotide substitutions in the first position of **G**GATCC hexamer. As shown in Fig. 2d, G > A and G > T substitutions increased the leakage, while G > C substitution allowed for a better growth in the presence of sucrose (Fig. 2c). Importantly, in all mutants, the ability to produce functional SacB was maintained as indicated by no growth upon induction. These conclusions were further confirmed by replacing *sacB* with *gfp*, what allowed for quantification of the leakage and induction levels (Fig. 2d). Although statistical analysis showed that difference between GGA and CGA variant is not significant, our results indicate that G > C substitution could decrease the leakage in some variants of the p_BAD_ promoter.

### Testing effects of an interfering sequence introduction

**Fig. 3:**
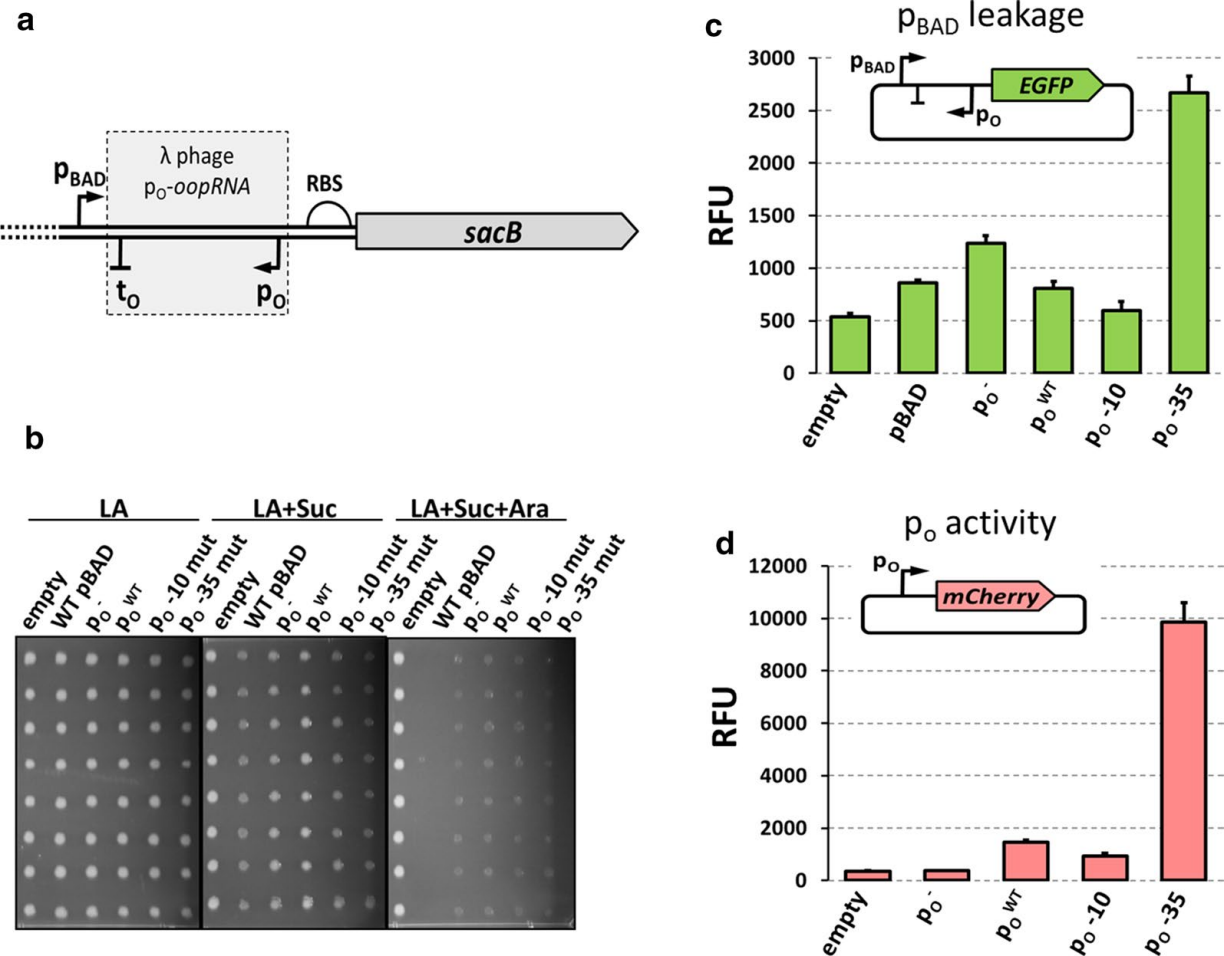
Introduction of the p_o_-*oopRNA* sequence in the 5′ UTR. **a** Schematic representation of construct tested with TECS, which contains p_O_-*oopRNA* fragment from bacteriophage lambda. **b** Growth of selected variants of p_O_-*oopRNA* region. Eight individual colonies were tested for each variant. **c** Background expression of p_O_-*oopRNA* variants measured by GFP fluorescence intensity and presented as relative fluorescence units. **d** The activity of mutated p_O_ promoters. Promoters were cloned into the pCh vector to drive the expression of *mCherry* gene

Next, we asked whether our protocol can be used to create an artificial regulatory system. The p_O_-*oopRNA* region from bacteriophage lambda was shown to interfere with transcription from the p_R_ promoter [34]. We tested whether this region can be used to control background expression from the p_BAD_ promoter by introducing the p_O_-*oopRNA* between the promoter and *sacB* RBS (Fig. 3a and Additional file 1: Figure S1a). As expected, the introduction of p_O_-*oopRNA* sequence had the suppressive effect on the leakage from the p_BAD_ promoter (Fig. 3b). Since it was proposed that this sequence exerts its regulatory effect through the transcriptional interference mechanism, we tested whether mutations altering the p_O_ promoter activity will influence the leakage of the p_BAD_ promoter (Fig. 3c, d). Surprisingly, we found that promoter strength does not correlate with inhibition of the p_BAD_ leakage. Nevertheless, these experiments show that our protocol can be successfully used to test influence of exogenous sequences on the activity of an inducible promoter.

### Optimization of the p_O_-*oopRNA*-mediated regulation

**Fig. 4:**
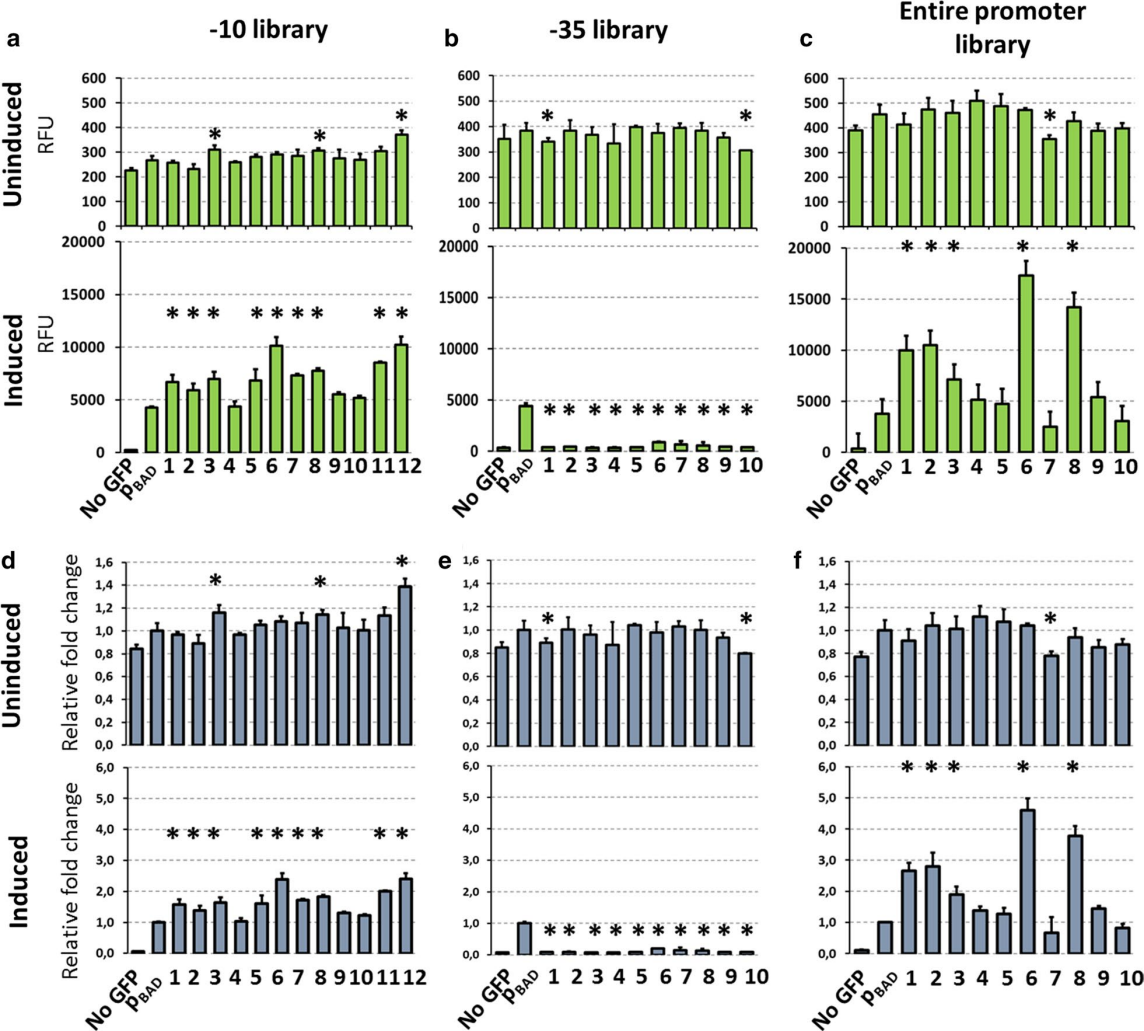
Effects of the p_O_ promoter sequence on the p_BAD_ promoter activity. **a**–**c** Activity of selected mutants from − 10 (**a**), − 35 (**b**) and whole promoter (**c**) libraries in LB medium, in the absence (upper panels) or presence of 0.1% arabinose (lower panels). **d**–**f** GFP fluorescence of each construct in respect to the wild type p_BAD_ promoter. Asterisks indicate significant differences (t test, *p *< 0.05)

Previous experiments showed that the wild-type p_O_-*oopRNA* sequence introduction results in the leakage comparable with the wild-type p_BAD_ promoter and that some mutations, namely − 10 mutant can reduce the leakage. Since rational design did not provide expected results, we used randomization of p_O_ promoter sequence in order to select mutants decreasing the leakage simultaneously allowing for efficient induction of the p_BAD_ promoter. Using PCR and oligonucleotides we introduced random sequence patch in − 10 box and − 35 box of the p_O_ promoter (Additional file 1: Figures S1b and S2). In addition, we used a vector amplification approach to introduce 35 nucleotides long random sequence in the p_O_ promoter region (Additional file 1: Figure S2). Shortly, 5′ phosphorylated oligonucleotides were used for whole vector amplification and the resulting linear product was self-ligated, generating circular plasmid with the randomized p_O_ promoter sequence (Additional file 1: Figure S1c). Eventually, we obtained three randomized promoter libraries (− 10, − 35 and entire promoter) which were screened with TECS. Selected mutants were used for the *gfp* expression assay as described previously (Fig. 4a, c). While the − 35 randomization generated mutants with impaired *gfp* expression upon induction, the other two libraries yielded mutants which had comparable leakage level but produced more GFP upon p_BAD_ induction with arabinose (Fig. 4d, e). Interestingly, properties of these mutants were maintained also in synthetic and rich medium indicating that observed effects are not dependent on the medium type (Additional file 1: Figure S3a, b).

**Fig. 5:**
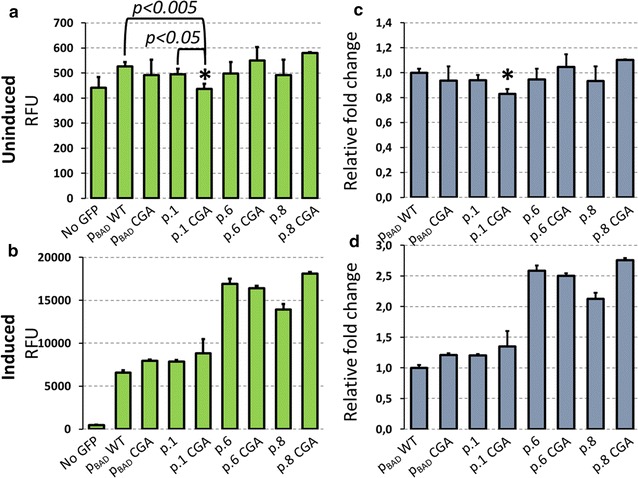
Combining mutations in p_BAD_ promoter and p_O_-*oopRNA*. **a**, **b** GFP fluorescence of plasmids p.1, p.6, p.8 from the entire promoter library and their variants with CGA mutation in *Bam*HI site. Fluorescence was measured in uninduced (**a**) and induced (**b**) cells. Bars represent average of three independent experiments and error bars show standard variation. Asterisk above bar indicates significant difference in fluorescence between p.1CGA, p.1 and the wild type p_BAD_ promoter. Significance was tested with t test and corresponding *p* values for comparisons are given. **c**, **d** Fluorescence of constructs in respect to the wild type p_BAD_ promoter

Next we asked whether combination of mutation in *Bam*HI and selected p_O_-*oopRNA* variants will result in improvement in the promoter regulation. For the experiment plasmids p.1, p.6 and p.8 from the entire promoter library were selected (Fig. 4). These constructs showed higher induction level and lower background transcription level than the wild type p_BAD_ promoter. In principle, combination of the G > C (CGA variant) mutation in the *Bam*HI site which decreased basal expression (Fig. 2d) with p_O_-*oopRNA* variants could result in improved promoters. However, experiments with CGA variants show that improvement was achieved only for the p.1 CGA variant, while p.6 CGA and p.8 CGA showed increased basal expression (Fig. 5a, b). Decreased basal expression in p.1 CGA was significantly lower than basal expression of the wild type p_BAD_ promoter and p.1 plasmid. While in p.1CGA, the G > C mutation and p_O_-*oopRNA* mutation showed synergistic effect, for other two p_O_-*oopRNA* combination affected background expression.

## Discussion

Despite the availability of a large number of promoter libraries, the development of novel, well-characterized and the most importantly, predictable promoters is one of the most urgent needs of synthetic biology [6]. However, the development of inducible promoters through randomization is complicated. It is likely due to the fact that in many cases interference with regulatory elements of an inducible promoter results in disruption of its functions [13, 24, 25]. Thus, attempts to optimize inducible promoters are laborious and time-consuming and in practice, the majority of obtained clones are characterized by increased leakage level in comparison to the initial promoter. To facilitate the process of inducible promoter optimization we present TECS, a strategy which allows for convenient optimization and modification of inducible promoters.

An important question would be why to optimize available inducible promoters at all? Inducible promoters are the most widely used in recombinant protein production where promoter activity is one of the key factors ensuring the high yield of the protein [3–5]. However, there is no universal promoter and specific project often require empirical testing of different systems or optimization. Furthermore, a subset of proteins is toxic to the host and expression of such targets requires the use of tightly regulated systems [35]. These needs drive the development of novel expression systems which ensure tight regulation of expression, satisfactory yield and possibly, ability to use in different hosts [10, 36, 37]. The majority if not all of the available inducible expression systems are derived from naturally occurring promoters and despite their modifications, these systems are based on promoters and functional elements present in bacteria or viruses. For that reason, these systems carry a burden of the intrinsic leakage level, a basal transcription in the absence of the inducer, which is present even in very tightly regulated p_BAD_ and *rhaT* promoters [10, 38]. This phenomenon is associated with stochastic gene expression observed in bacterial populations and in the course of evolution it increases survival chances in rapidly changing environments [39]. Therefore it is expected that in contrast to needs of synthetic biology and biotechnology, naturally occurring inducible promoters evolved to allow some level of background expression in the absence of the inducer. However, as shown by our results, by using TECS it is possible to select for synthetic inducible promoters which may meet the expectations of the aforementioned disciplines.

Conceptually, TECS is a method of evolving inducible promoters in an artificial environment designed to select desired features, low leakage and high induction level. The alternative description of TECS could be that it is a two-factor discrimination test. The first factor is the basal expression of tested promoter (leakage) and high-leakage variants are counter-selected in the first step where they are unable to grow on sucrose. The second factor is the induction, since inducible promoter should be activated by specific metabolite or conditions. Thus the second step of selection tests promoter induction and ability to produce toxin by selected mutants. This simple strategy allows TECS to select potentially interesting mutants from randomized libraries. Cells harbouring a leaky promoter will be unable to grow on sucrose-containing medium and the use of solid medium ensures the stringent selection for plasmids [35]. In the second step, individual clones are verified for the leakiness and for production of functional SacB upon induction. Overall, the selection process is carried out with simple microbiological procedures and does not require complex equipment.

In our opinion TECS fills a gap in available methods for promoter libraries generation. Computational methods which allow for in silico design of promoters with different strength, despite their increasing accuracy often yield unpredictable results [6]. It is important to remember that computational methods are developed on the basis of available experimental data [18, 20]. Thus although their accuracy and power is constantly growing, they are limited by availability of experiments. Furthermore, prediction of effects of individual mutations or their combinations could be inaccurate not only for computational methods. In this work we show that even prediction of effects for mutants selected with TECS could yield unpredictable results (Fig. 5). Thus it is likely that even the most robust computational methods will require extensive experimental verification of proposed promoters. While TECS would not be the best choice for verification of the in silico design, it is an excellent method to generate a high number of promoter variants, namely the training set for computational methods. In our opinion, TECS is more convenient than available promoter engineering methods which are based on a fluorescent protein expression and require a fluorescence activated cell sorter (FACS). These methods were used to prepare libraries of constitutive promoters or inducible promoters [15, 22, 23]. In comparison with FACS-based methods, TECS certainly has lower throughput but it is compensated by the high success rate of improved promoters’ selection.

One of the best arguments supporting the usefulness of TECS is improving the p_BAD_ promoter, which was not changed since its introduction into expression vectors [32]. We were able to eliminate the inconvenient *Bam*HI site by mutagenesis, despite previous reports that insertions and deletions in this affect the leakage and activity of the promoter [13]. Interestingly, although changes in the leakage level of created variants were relatively small, TECS was sensitive enough to detect them (Fig. 1d and e). In our experiments we used a tightly regulated p_BAD_ promoter. However, TECS could be used to optimize any inducible promoter. In this work we present two convenient methods for randomization of particular DNA sequence. The first method utilizes a well described mutagenesis with primer containing a random sequence patch (Additional file 1: Figure S1b). However, for introduction of long random sequence mutagenesis approach in our experience was ineffective (low yield of mutants). Thus we developed alternative protocol using two phosphorylated oligonucleotides with randomized 5′ end to amplify the vector (whole vector amplification, Additional file 1: Figure S1c). Using PCR with novel generation of fast and high fidelity DNA polymerases it is possible to generate a linear vector molecule with random sequence at its end. We observed that this procedure is very effective in construction of random mutants of a p_O_ promoter sequence. However, we are aware that in our experiments we tested only a minor subset of possible combinations. In theory, 35 nucleotides long random sequence generates over 10^21^ combinations. In order to achieve onefold coverage it would be necessary to generate over 10^21^ vector molecules and corresponding amount of primers would be 1.66 mmol of each, that is over 10^9^ times more molecules that are used in a standard PCR. Nevertheless, despite the fact that we covered merely a pinch of possible combinations, TECS allowed for selection of mutants which were better than the initial vector.

## Conclusions

In this work, we described TECS as a novel tool for optimization of inducible expression systems in *E. coli*. We show that TECS allows for convenient, in vivo selection of mutants with desired properties. Although the method was tested on the well-studied p_BAD_ promoter, it can be directly applied to modify any system. Optimization with TECS can be performed in any microbiological laboratory, using the basic equipment. The versatility and simplicity of the method make it an attractive choice for the generation of in-house-optimized vectors for controlled gene expression.

## Additional file

**Additional file 1.** Additional Tables S1, S2; Figures S1–S3.

## Abbreviations

cAMP: cyclic adenosine monophosphate
FACS: fluorescence-activated cell sorting
PCR: polymerase chain reaction
