# Supplementary material for: TECS: a toxin expression control strategy as a tool for optimization of inducible promoters
Source: Microb Cell Fact. 2018 Mar 13;17:40. doi: 10.1186/s12934-018-0891-1 (PMC5851080; doi:10.1186/s12934-018-0891-1)
Supplement: Supplementary file 1 — Additional file 1. Additional Tables S1, S2; Figures S1–S3. [file 12934_2018_891_MOESM1_ESM.docx]

**Additional data**

TECS: a toxin expression control strategy for optimization of inducible promoters.

Aleksandra Małachowska^1^ and Paweł Olszewski^1^*

^1^Department of Genetics and Biosystematics, University of Gdańsk, ul. Wita Stwosza 59, 80-308 Gdańsk, Poland.

*correspondence to Paweł Olszewski

Phone: +48 585 236 098; e-mail: [pawel.olszewski@biol.ug.edu.pl](mailto:pawel.olszewski@biol.ug.edu.pl)

**Additional tables**

**Table S1Plasmids used in this study.**

| Plasmid | Genotype or characteristics |
| --- | --- |
| pBAD24 | Cloning vector with arabinose-regulated pBAD promoter, pBR322 ori and ampicilin resistance marker (bla). |
| pBAD24cm | As pBAD24 but bla coding sequence was replaced with cat coding sequence (chloramphenicol resistance marker). |
| pBAD-sacB | As pBAD24cm but sacB coding sequence was introduced in-frame, under control of pBAD promoter. |
| pBAD-poop | As pBAD-sacB but pO-oopRNA fragment of lambda phage was introduced between pBAD TSS and RBS. |
| pBAD-GFP | As pBAD-sacB but sacB CDS was replaced with GFP CDS. |
| pBAD-poop-GFP | As pBAD-poop but sacB CDS was replaced with GFP CDS. |

**Table S2 Primers used in this study.**

| **Primer name** | **Sequence (5' to 3')** | **Description** |
| --- | --- | --- |
| oligo1 | CAACTCTCTACTGTTTCTCCTAAAAAACGCCCGGCGGCAACCGAGCG | construction of  pO-oopRNA insert |
| oligo2 | TGACCTCAGAACTCCATCTGGATTTGTTCAGAACGCTCGGTTGCCGCC |  |
| oligo3 | ACTCCTGTTGATAGATCCAGTAATGACCTCAGAACTCCATC |  |
| oligo4 | GAGTATTTTTGCTGTATTTGTCATAATGACTCCTGTTGATAGATCC |  |
| oligo5 | GTTCATGGTGAATTCCTCCTTTGAGTATTTTTGCTGTATTTGT |  |
| gfp2bad_up | GGAGGAATTCACCATGAACATCAAAGTGAGCAAGGGCGAGGAGC | Replacement of *sacB* with *gfp* |
| gfp2bad_down | CAGGCTGAAAATCTTCTCTCATTACTTGTACAGCTCGTCCATGCCG |  |
| sac2bad_fw | GGGCTAGCAGGAGGAATTCACCATGAACATCAAAAAGTTTGCAAAAC | *sacB* insertion into pBAD24 |
| sac2bad_rev | TATCAGGCTGAAAATCTTCTCTCATTATTTGTTAACTGTTAATTGTCC |  |
| cat_overlap_fw | GCTTCAATAATATTGAAAAAGGAAGAGTATGGAGAAAAAAATCACTGG | *cat* insertion into pBAD24 (Cm^R^) |
| cat_overlap_rev | TATATATGAGTAAACTTGGTCTGACAGTTATTACGCCCCGCCCTGCC |  |
| pomut1(P) | NNNNNNNNNNNNNNNNNNNNAGGAGGAATTCACCATGAACATCAAAAAGTTTGC | p_O_ promoter randomization |
| pomut2(P) | NNNNNNNNNNNNNNNNGTTGATAGATCCAGTAATGACCTC |  |
| 10_mut | GTGAGGGGGCTGGNNNNNNCACATATGTTGATAGATCCAG | -10 hexamer randomization |
| 10_rev | CCAGCCCCCTCACTACATGTC |  |
| 35_mut | CATGGTGAATTCCTCCTNNNNNNTGTAGTGAGGGGGCTGG | -35 hexamer randomization |
| 35_rev | AGGAGGAATTCACCATGAACATCAAAAAG |  |
| pBAD_seq2 | CTAACCAAACCGGTAACCCCGC | sequencing primer |

**Additional figures**


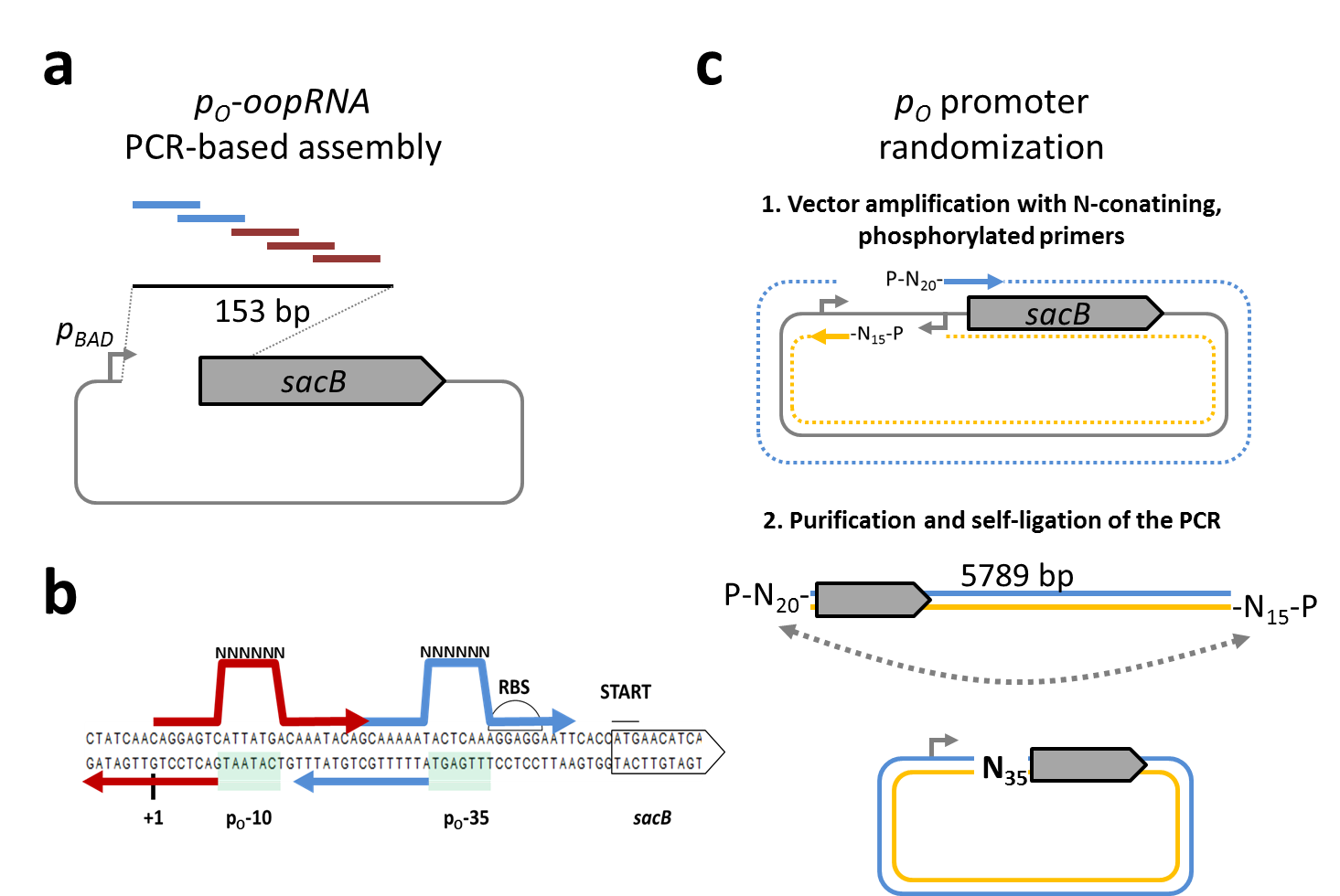


**Figure S1. Cloning strategies used for construction of p_O_ promoter variants.** a) PCR-based assembly of the p_O_-oopRNA fragment basing on the sequence with accession number J02459.1. b) Schematic representation of randomization strategy for -10 and -35 regions of the p_O_ promoter. c) Schematic representation of p_O_ promoter sequence randomization. The entire vector was amplified with phosphorylated primers containing 15 and 20 random positions at the 5’ end. Obtained PCR product was used for circularization with T4 DNA ligase.


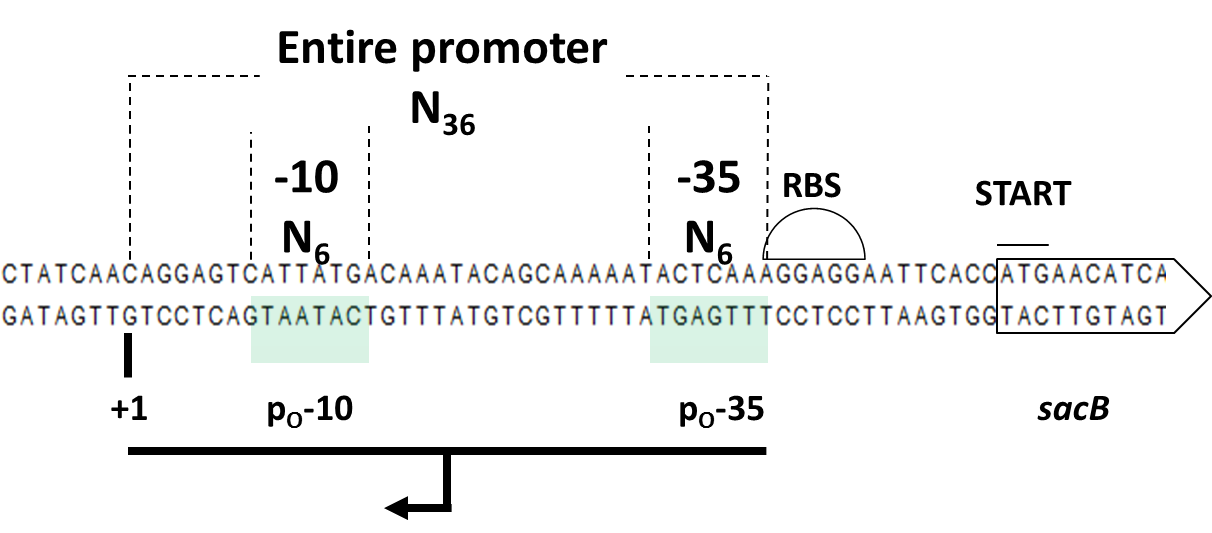


**Figure S2. Schematic representation of p_O_-oopRNA regions randomized for TECS.** Three different libraries were created by randomization of -10 and -35 hexamers as well as the whole promoter sequence (from -1 to -35). Mutations do not affect the RBS nor the start codon.


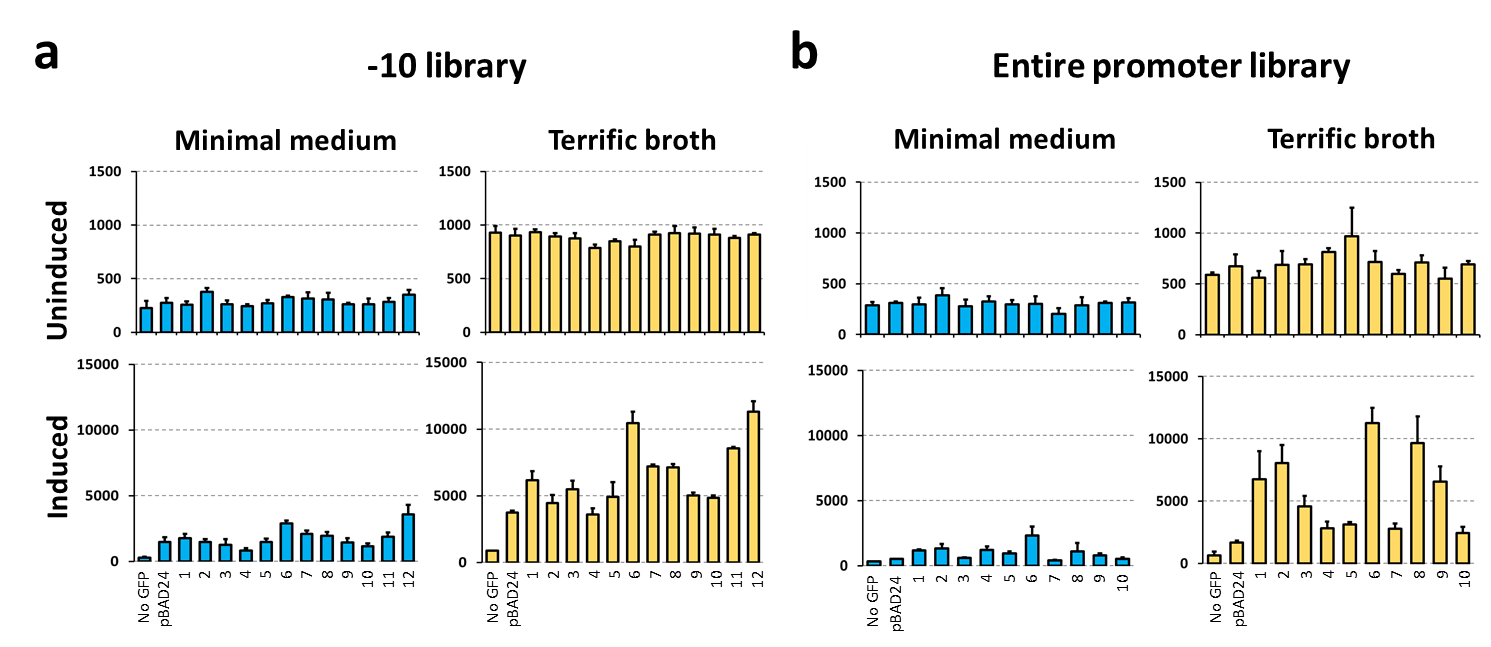


**Figure S3 Regulation of pBAD-p_O_-*oopRNA* clones in different media.** Clones expressing GFP from -10 library (a) and the entire promoter library (b) were grown in minimal media (blue bars) or terrific broth (yellow bars). Charts represent relative GFP fluorescence measured as ratio of GFP fluorescence to the culture density. For both libraries GFP fluorescence was measured before induction (upper panels) and after 180 min of induction with 0.2% arabinose. Bars represent average of three independent measurements, standard deviation is indicated.
